# Discovery of a novel antibacterial protein CB6-C to target methicillin-resistant *Staphylococcus aureus*

**DOI:** 10.1186/s12934-021-01726-9

**Published:** 2022-01-04

**Authors:** Haipeng Zhang, Jingrui Chen, Yuehua Liu, Qijun Xu, Muhammad Inam, Chengguang He, Xiuyun Jiang, Yu Jia, Hongxia Ma, Lingcong Kong

**Affiliations:** 1 College of Life Science, Jilin Agricultural University, Xincheng Street No. 2888, Changchun, 130118 China; 2 The Engineering Research Center of Bioreactor and Drug Development, Ministry of Education, Jilin Agricultural University, Xincheng Street No. 2888, Changchun, 130118 China; 3 College of Veterinary Medicine, Jilin Agricultural University, Xincheng Street No. 2888, Changchun, 130118 China; 4 The Key Laboratory of New Veterinary Drug Research and Development of Jilin Province, Jilin Agricultural University, Xincheng Street No. 2888, Changchun, 130118 China; 5 Changchun Sci-Tech University, Shuangyang District, Changchun, 130600 China

**Keywords:** *Bacillus velezensis* CB6, MRSA, Antibacterial protein, Characterization, Mechanistic

## Abstract

Given a serious threat of multidrug-resistant bacterial pathogens to global healthcare, there is an urgent need to find effective antibacterial compounds to treat drug-resistant bacterial infections. In our previous studies, *Bacillus velezensis* CB6 with broad-spectrum antibacterial activity was obtained from the soil of Changbaishan, China. In this study, with methicillin-resistant *Staphylococcus aureus* as an indicator bacterium, an antibacterial protein was purified by ammonium sulfate precipitation, Sephadex G-75 column, QAE-Sephadex A 25 column and RP-HPLC, which demonstrated a molecular weight of 31.405 kDa by SDS-PAGE. LC–MS/MS analysis indicated that the compound was an antibacterial protein CB6-C, which had 88.5% identity with chitosanase (Csn) produced by *Bacillus subtilis* 168. An antibacterial protein CB6-C showed an effective antimicrobial activity against gram-positive bacteria (in particular, the MIC for MRSA was 16 μg/mL), low toxicity, thermostability, stability in different organic reagents and pH values, and an additive effect with conventionally used antibiotics. Mechanistic studies showed that an antibacterial protein CB6-C exerted anti-MRSA activity through destruction of lipoteichoic acid (LTA) on the cell wall. In addition, an antibacterial protein CB6-C was efficient in preventing MRSA infections in in vivo models. In conclusion, this protein CB6-C is a newly discovered antibacterial protein and has the potential to become an effective antibacterial agent due to its high therapeutic index, safety, nontoxicity and great stability.

**Supplementary Information:**

The online version contains supplementary material available at 10.1186/s12934-021-01726-9.

## Introduction

Multidrug-resistant bacteria, such as methicillin-resistant *Staphylococcus aureus* (MRSA) etc. are remarkable foodborne bacteria of nosocomial and community-acquired bacterial infections around the world and have been spreading at alarming rates globally [1]. Once humans and animals are infected with MRSA, it can cause a variety of diseases, including food poisoning, abscesses, skin infections, pneumonia, septicemia and it can even lead to death [2]. This poses a serious threat to human health and food safety [3]. Currently, eradication of the spread of such pathogens is very difficult. Therefore, it is need of the day to discover new antibacterial agents with highly effective antibacterial activity and safety for combating MRSA.

In previous studies, it has been found that bacteria can produce many antibacterial drugs for treating various diseases, and some newly discovered bacteria produce effective antibacterial activities. These new bacteria have attracted researchers to study which molecule is responsible for the main antibacterial activity [4].

In our previous experiment, we had isolated *Bacillus velezensis* CB6 from the soil of Changbaishan, China which had effectively inhibited MRSA. Through 40% ammonium sulfate precipitation, purification and mass spectrometry identification, we had purified a protein with an antibacterial activity against MRSA. However, recent study discovered that crude protein precipitated with 100% saturated ammonium sulfate leads to a better antibacterial effect against MRSA. Therefore, in this study, the crude protein from *B. velezensis* CB6 was precipitated with 100% saturated ammonium sulfate. A series of protein purification methods were used to identify the antibacterial protein as antibacterial protein CB6-C, which was shown to be stable and safe against MRSA and exhibited effective antibacterial activity both in vitro and in vivo. These characteristics make the antibacterial protein CB6-C more likely to become a potential candidate as an antibacterial agent.

## Materials and methods

### Bacterial cultures, media and ethical considerations

All bacterial strains, culture conditions and sources used in this study are listed in Additional file 1: Table S1. RAW 264.7 cells were obtained from the College of Animal Medicine, Jilin Agricultural University Changchun, China. Luria–Bertani broth medium (LB) and Luria–Bertani agar medium (LBA) were purchased from Sangon Biotech (Shanghai, China), and Mueller–Hinton broth medium (MHB) was purchased from GL Biochem (Shanghai, China). Mice (weighing 16–20 g) were purchased from the Experimental Animal Center of Jilin University (Changchun-Jilin, China). All mice tests were carried out in accordance with the Regulations for Animal Experimentation at Jilin Agricultural University (JLAU08201409) and Laboratory Animal Care and Use Guidelines of National Institutes of Health (NIH Publication No. 8023).

### Purification of the crude protein

According to the previously described methods of our laboratory [5], antibacterial protein CB6-C was purified through 100% ammonium sulfate precipitation, Sephadex G-75 column, QAE-Sephadex A 25 column and chromatography C4 column (5 μm, 4.6 mm × 250 mm, Agilent, USA). Briefly, purified *B. velezensis* CB6 was inoculated into 500 mL LB broth medium at 1% (v/v) and shaken at 37 °C for 48 h. The cultures were centrifuged at 4 °C and 8000 × g for 60 min, and then the supernatants were filtered twice through 0.22-μm membranes (BioLeaf, Shanghai, China) to remove cells. Next, ammonium sulfate was gently added to the collected supernatant to reach 100% saturation, stirred at 4 °C for 12 h, and allowed to stand overnight followed by centrifugation (12,000×*g*, 30 min, 4 °C). The sediment was resuspended in 10 mM phosphate-buffered saline (PBS, pH 7.4), and a 10 KD ultrafiltration tube was used to remove ammonium sulfate and small molecule compounds. The samples containing proteins larger than 10 KD were tested for antibacterial activity. The active samples were loaded onto a Sephadex G-75 (3 KD–70 KD, Sigma, USA) gel filtration column, connected to an AKTA purification system (Boston, USA) and eluted at 1 mL/min flow rate. The proteins were classified according to the different molecular weights, and the separated peaks were collected at 280 nm and tested for antibacterial activity. The collected antibacterial fraction was further purified by a QAE-Sephadex A 25 column. Different concentrations of NaCl (0, 0.1 M, 0.2 M and 0.3 M) in 100 mM Phosphate buffer (pH 7.0) were used to elute the antibacterial protein. Next, for further purification of the crude antibacterial proteins, active samples were applied to a reverse-phase high-performance liquid chromatography system (RP-HPLC, Agilent, CA, USA) equipped with a reverse-phase chromatography C4 column. Elution was performed by using a 5–90% linear gradient of acetonitrile containing 1% trifluoroacetic acid with 1 mL/min flow rate for 60 min. The separate peaks of the UV detector were recorded at 280 nm. For the above test, after each purification step, MRSA was used as indicator bacteria, and the agar diffusion method was used to determine the antibacterial activity [6].

### Molecular mass determination and LC–MS/MS analysis of antibacterial protein CB6-C

After purification of the protein using a C4 column, partially active samples were applied to a 12% polyacrylamide gel for sodium dodecyl sulfate polyacrylamide gel electrophoresis (SDS-PAGE, Mini-PROTEAN II Electrophoresis Cell, Bio-Rad Laboratories, USA) and subjected to a 100 V constant voltage for separation. Then, the gel was stained with Coomassie brilliant blue and washed three times with Phosphate buffer to remove impurities, and the molecular size of the purified antibacterial protein was determined. Subsequently, only a single band was cut and sent to gene sequencing company (Wuhan, China) to determine the protein sequence by using liquid chromatography-tandem mass spectrometry (LC–MS/MS, Bruker Daltonics, Germany).

### Safety assays

The hemolytic activity of the antibacterial protein CB6-C was examined according to a protocol as described previously [7]. Briefly, the collected sheep blood cells were washed with PBS (pH 7.4) three times and diluted to 2% in PBS. Then, equal volumes of sheep blood cells were mixed with different concentrations of antibacterial protein CB6-C (0.5 µg/mL to 256 µg/mL) in tubes and incubated at 37 °C for 1 h. Subsequently, the mixtures were centrifuged at 1000 × g for 10 min, and the supernatants were transferred to 96-well plates. Equal amounts of PBS and 0.2% Triton X-100 were added as negative and positive controls, respectively. The absorbance of the mixtures was measured by the OD at 570 nm. The experiment was performed three times. RAW 264.7 cells were used to study the cytotoxicity of the antibacterial protein CB6-C. This test used the method described by Xu et al. [8]. In brief, RAW 264.7 cells were collected and washed twice with DMEM, and equal amounts of the cells were placed into 96-well plates at a density of 10^5^ cells per milliliter, and cultured overnight at 37 °C under condition of 5% CO_2_. Then, equal amounts of antibacterial protein CB6-C were mixed with the cells, and the final concentrations of antibacterial protein CB6-C per well were 0.5 µg/mL to 128 µg/mL. After 16 h of culture at 37 °C, CCK-8 (10%, v/v) was added to each mixed cell’s well in the 96-well plates and cultured at 37 °C for 2 h. The absorbance was measured by the OD at 450 nm (microplate reader, TECAN GENios F129004, Tecan, Salzburg, Austria). The experiment was performed three times.

### Antibacterial spectrum and minimum inhibitory concentration

The method described by Jia et al. [9] was adopted to determine the antibacterial spectrum and minimum inhibitory concentration (MIC) of antibacterial protein CB6-C. In short, the antibacterial protein CB6-C collected from RP-HPLC was condensed into powder. The powder was dissolved with PBS until reaching a concentration of 512 µg/mL and added to the first well in each row in 96-well plates, and multiple dilutions with PBS were performed at final concentrations ranging from 0.5 to 256 µg/mL. Concurrently, various indictor strains were inoculated into MHB broth medium and shaken at 37 °C and 180 rpm to cultivate to log phase growth (OD at 600 nm = 0.5). Then, the bacterial concentration was adjusted to approximately 10^5^ CFU/mL using MHB medium, and an equal volume of bacteria was added to 96-well plates. After incubating at 37 °C for 18 h, the absorbance was measured by the OD at 600 nm (microplate reader, TECAN GENios F129004, Tecan, Salzburg, Austria). The MIC was defined as the lowest concentration with no growth of bacteria after incubation at 37 °C for 16–20 h.

### Time-kill kinetics assay

The time-kill kinetics of MRSA were determined as previously described [10]. In brief, the MRSA cells at the logarithmic growth phase were adjusted to an OD of 0.5 at 600 nm, and the bacterial concentration of approximately 10^5^ CFU/mL was obtained using MHB broth medium. Next, antibacterial protein CB6-C was added to a final concentrations of 1 × MIC, 2 × MIC and 4 × MIC, with an equal volume of PBS added to antibacterial protein CB6-C as a control followed by shaking at 37 °C and 180 rpm for 24 h. During this period, equivalent amounts of liquid were taken every four hours for serial tenfold dilutions, and then a 100 µl of the dilutions was spread on the LB plate, after which the colonies were counted. The experiment was performed three times.

### Stability assays

The purified antibacterial protein CB6-C was used to test the temperature stability according to the method described by Tumbarski et al. [11]. Equivalent amounts of antibacterial protein CB6-C were put in the test tubes, sealed and then placed at 40 °C, 50 °C, 60 °C, 70 °C, 80 °C, 90 °C and 100 °C for 60 min and 121 °C (autoclaving for 15 min). To determine the effect of enzymes on the antibacterial activity of antibacterial protein CB6-C, a final concentration of 1 mg/mL of catalase (5000 µ/mg), pepsin (250 µ/mg), papain (800 µ/mg), trypsin (10,000 µ/mg) and proteinase-K (30 µ/mg) manufactured in Sigma-Aldrich, (Merck, USA) was added to equivalent amounts of antibacterial protein CB6-C samples. After incubation at 37 °C for 30 min, the enzyme reaction was stopped by heating at 100 °C for 10 min. To assess the influence of acid bases on antibacterial protein CB6-C activity, equivalent amounts of antibacterial protein CB6-C were adjusted to pH 2–12 by using HCl and NaOH and incubated at 37 °C for 1 h. Subsequently, all antibacterial protein CB6-C samples were readjusted to pH 7.0. To test the organic reagent effect on antibacterial protein CB6-C activity, 1% (v/v) methanol, isopropanol, Tween 20, Tween 80, acetonitrile, acetone and EDTA (Sigma-Aldrich, Merck) were incubated with antibacterial protein CB6-C at 37 °C for 1 h.

For the experiment outlined above, untreated antibacterial protein CB6-C was used as a positive control, and the inhibition zones of the treated antibacterial protein CB6-C and positive control samples were measured to estimate the influencing factors of the antibacterial protein CB6-C. Each experiment was repeated three times.

### Synergy with conventional antibiotics

The antibacterial effects of antibacterial protein CB6-C in combination with other antibiotics were evaluated by checkerboard tests [12]. In short, antibacterial protein CB6-C and antibiotics were prepared at final concentrations from 1 × MIC to 1/64 × MIC. Next, the same concentration of antibacterial protein CB6-C was added to the horizontal row of 96-well plates, and the same concentration of antibiotic was added to the longitudinal column of 96-well plates. Then, a 10^5^ CFU/mL MRSA was added to each well and incubated at 37 °C for 10 h. Each test was performed three times. The fractional inhibitory concentration (FIC) index was calculated as follows:

$${\text{FIC}}\, = \,\left( {{{\text{MIC of A in combination}} \mathord{\left/ {\vphantom {{\text{MIC of A in combination}} {\text{MIC of A alone}}}} \right. \kern-\nulldelimiterspace} {\text{MIC of A alone}}}} \right) + \,\left( {{{\text{MIC of B in combination}} \mathord{\left/ {\vphantom {{\text{MIC of B in combination}} {\text{MIC of B alone}}}} \right. \kern-\nulldelimiterspace} {\text{MIC of B alone}}}} \right),$$

where FIC ≤ 0.5 denoted synergy and 0.5 < FIC ≤ 1.0 denoted an additive effect.

### Effect of metal ion on CB6-C activity

Effects of metal ions (K^+^, Co^2+^, Ni^+^, Mg^2+^, Mn^2+^, Ca^2+^, Ba^2+^, Fe^3+^ and Cu^2+^) on CB6-C antibacterial activity were carried out according to the protocol described previously [13]. Briefly, a 512 ug/mL of CB6-C was continuously twofold diluted to 1ug/mL and added metal ions to CB6-C diluents of different concentrations to make a final concentration of metal ions in each of a 10 mM. Then, a 50 uL metal ions diluent was added to the 96-well plate as test group, respectively. Additionally, we used PBS to dilute metal ions to a 10 mM and took a 50 uL diluted liquid and added to the 96-well plate as a negative control. Then, a 50 uL MRSA (10^5^ CFU/mL) was added to each well and incubated at 37 °C for 18 h. The absorbance was measured by the OD at 600 nm and the minimum growth concentration was noted for evaluating bacterial growth. The experiment was performed three times.

### Measurement of ROS release

The total reactive oxygen species (ROS) released from MRSA treated with the different concentrations of antibacterial protein CB6-C (8 µg/mL, 16 µg/mL, 32 µg/mL and 64 µg/mL) were probed with an ROS assay kit (Nanjing Jiancheng Bioengineering Institute, Jiangsu, China), and performed according to the manufacturer's instructions. No treatment with antibacterial protein CB6-C was performed as the negative control for ROS production. The fluorescence value of each sample was measured with an F4500 fluorescence spectrophotometer (emission λ = 525 nm, excitation λ = 488 nm) (Hitachi, Tokyo, Japan).

### Measurement of adenosine triphosphate (ATP) and alkaline phosphatase (AKP) release

Intracellular ATP and AKP leak out when bacteria are destroyed. Therefore, we examined the amount of extracellular AKP and intracellular ATP to evaluate the effect of antibacterial protein CB6-C on MRSA [14, 15]. In brief, after the MRSA strain was cultured to logarithmic growth phase (OD at 600 nm = 0.5), it was centrifuged and washed twice with PBS (pH 7.4). The washed MRSA cells were treated with 1 × MIC of antibacterial protein CB6-C and incubated at 37 °C for 6 h, and the amount of extracellular AKP release was examined every hour. Similarly, MRSA cells were treated with 8 µg/mL to 64 µg/mL of antibacterial protein CB6-C for 1 h to examine the amount of intracellular ATP. The amount of extracellular AKP and intracellular ATP was measured using the ATP and AKP test kits (Jiancheng Biology Engineering Institute, Nanjingjiancheng, China) according to the manufacturer's instructions.

### Membrane permeability assay

Changes in membrane permeabilization were determined by measuring intracellular β-galactosidase activity as previously described. In short, MRSA in the mid-log phase was washed with PBS three times, diluted to 10^5^ CFU/mL and an equal amount was added to a 96-well plate. Concurrently, different concentrations of antibacterial protein CB6-C (1 × MIC to 4 × MIC) were added to each well and incubated at 37 °C. In addition, the antimicrobial peptide LR18 (it has been reported to destroy MRSA cell membranes) stored in our laboratory was adjusted to a final concentration of 1 × MIC as a positive control [9]. The absorbance was measured by the OD at 420 nm and recorded for 1 h after every 10 min.

### Preparation of protoplasts and MIC assay

The preparation of protoplasts was carried out according to the method of Fan et al. [16]. In short, MRSA cells were cultured on MH broth medium to the logarithmic growth phase (OD at 600 nm = 0.5). A 5 mL of MRSA bacterial suspension was mixed with 5 mL of lysozyme (100 μg/mL) and incubated at 37 °C for 1 h. Then, the bacterial suspension was centrifuged at 4000 × g for 10 min at 4 °C, and the supernatant was discarded. Two washes with hypertonic buffer (0.1 mol/L phosphate buffer pH 6.0 and 0.8 mol/L Mannitol) were used to remove the enzyme, and protoplasts were suspended in a 5 mL of hypertonic buffer. The protoplast detachment was immediately observed under the microscope. The preparation of protoplasts was considered successful when more than 95% of the cells were gram-stained red. In addition, to evaluate the effect of antibacterial protein CB6-C on the cell membrane, MIC tests were performed on the prepared protoplasts according to the method outlined above in “Antibacterial spectrum and minimum inhibitory concentration” section. (MIC test methods), and untreated MRSA was used as the positive control.

### Scanning and transmission electron microscopy

To investigate the morphological changes of MRSA cells after treatment with antibacterial protein CB6-C, we performed Scanning electron microscopy (SEM) according to a method outlined in a previous study [17]. In brief, MRSA of logarithmic growth phase (OD at 600 nm = 0.5) was diluted to 10^5^ CFU/mL, added to a final concentration of 16 μg/mL antibacterial protein CB6-C (1 × MIC), and then incubated in the 6-well cell plate (containing polylysine-treated glass slides) at 37 °C for 3 h, with untreated MRSA cells used as a control. After incubation, the bacterial suspension was removed, and the polylysine-treated glass slides were fixed with 2.5% glutaraldehyde at 4 °C for 12 h, dehydrated with ethanol dilutions, dried and sprayed. The bacterial specimens were imaged using a FlexSEM 1000 SEM (JEOL, Hitachi, Tokyo, Japan).

For clearer observation of intracellular changes in MRSA, transmission electron microscopy (TEM) was performed according to the protocol described by Qin et al. [18] Briefly, cells were fixed with 2.5% glutaraldehyde as described above. After that, cells were osmicated in 2% osmium tetroxide for 4 h, dehydrated with ethanol solutions and embedded in epoxy resin. Finally, the sections were coated and stained using 2% uranyl acetate and lead citrate and observed with an E-1010 TEM (JEOL, Hitachi, Tokyo, Japan).

### Competitive inhibition assay

To detect the effect of CB6-C on the cell wall main components (peptidoglycan, membrane teichoic acid, and Staphylococcal Protein A) of *Staphylococcus aureus* (*S. aureus*), CB6-C was diluted to different concentrations (4 µg/mL to 512 µg/mL), and the MIC assay was performed as described above in “Antibacterial spectrum and minimum inhibitory concentration” section. (MIC test methods). During the test, a 10 µg of equal volume of peptidoglycan, membrane teichoic acid, and Staphylococcal Protein A from *S. aureus* was added to each well of the 96-well plate to detect the effect of different proteins on the antibacterial activity of CB6-C.

### Mouse infection models

To determine the treatment effect of antibacterial protein CB6-C in mice, we adopted the method based on previous studies by Song et al. [19] Briefly, a total of 30 BALB/c female mice were randomly transferred to three groups of cages (n = 10 per group), and each mouse was infected with a dose of 1.15 × 10^9^ CFU MRSA in suspension via intraperitoneal injection (previous experimental results showed that the mortality rate of mice infected with this dose was higher than 80% within 48 h). The mice were treated with a specified intraperitoneal administration of PBS, antibacterial protein CB6-C (10 mg kg − 1), or antibacterial protein CB6-C (20 mg kg − 1) after one hour infection. The mice were observed for 48 h, and dead mice were removed to confirm the treatment effect of antibacterial protein CB6-C.

The mouse organ bacterial load test was the same as the treatment trial method, 40 mice were randomly divided into two groups (n = 20 per group) and infected intraperitoneally with 1.15 × 10^9^ CFU MRSA in suspension. At 1 h postinfection, mice were treated with PBS and antibacterial protein CB6-C (20 mg kg − 1). After 48 h, the mice were euthanized, the heart, liver, spleen, lungs and kidneys were removed, and one part was washed with sterile PBS, soaked in 4% paraformaldehyde for fixation, and waited for subsequent hematoxylin–eosin (HE) staining. Other part of the organs tissue was washed with sterile PBS and homogenized in sterile PBS, and the bacterial loads in the different organs were counted.

### Statistical analysis

One-way analysis of variance (ANOVA) was performed using SPSS v.22.0 software, followed by Tukey test. All data results were expressed as mean ± standard deviation. The mean values were considered significant different when *p < 0.05, **P < 0.01.

## Results

### Purification and identification of antibacterial proteins

Antibacterial crude proteins present in the supernatant were collected by precipitation with 100% saturated ammonium sulfate, and crude proteins were filtered twice through a 10 kDa ultrafiltration tube. After that, antibacterial crude proteins were loaded onto a Sephadex G-75 gel filtration column using an AKTA purification system. As shown in Additional file 2: Table S Fig S1A, only one single peak was collected, and an antibacterial test showed that this peak had high antibacterial activity.(data not shown). For further purification of antibacterial proteins, we loaded the obtained crude antibacterial proteins onto a QAE-Sephadex A 25 column, and four peaks were collected. Among them, only the third peak had antibacterial activity (Additional file 2: Fig. S1B). Proteins with antibacterial activity were loaded onto a C4 column using RP-HPLC. Some separate peaks were observed by measuring the absorbance at 280 nm using an UV detector. Among these, only one peak had antibacterial activity, and the other peaks did not show antibacterial activity (Additional file 2: Fig S2A). The single peak antibacterial protein was collected and assessed by SDS-PAGE. The results revealed a single monomeric protein band with a molecular mass estimated to be approximately 31 kDa (Additional file 2: Fig S2B). It was obvious that the protein had high purity and could be identified by LC–MS/MS. By LC–MS/MS analysis and comparison with the UniProt database (https://www.uniprot.org), the protein showed 88.5% identity to the chitosanase (Csn) protein of *Bacillus subtilis* 168 (Entry: O07921). The target protein consisted of 278 amino acids with an isoelectric point of 8.65 and a molecular mass of 31.405 kDa. The apparent molecular mass of the target protein and the SDS-PAGE results showed that the measurements by the LC–MS/MS were almost the same. Therefore, the purified antibacterial protein was initially determined to be chitosanase, and we designated it an antibacterial protein CB6-C.

### Safety assays

**Fig. 1:**
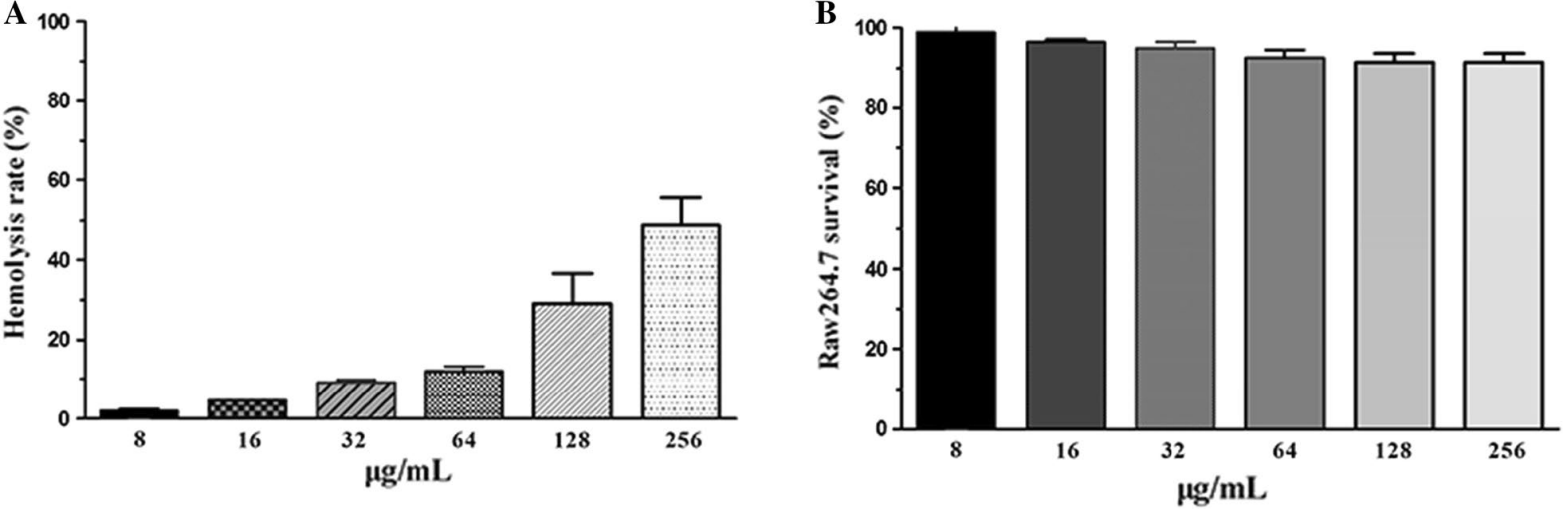
Safety assays of CB6-C on red blood cells and animal cell. **A** Hemolytic activity of CB6-C to the sheep red blood cells. **B** Cytotoxicity of CB6-C against RAW 264.7 cells

The hemolytic activity of the antibacterial protein CB6-C was assessed by measuring its ability to lyse sheep blood cells at different concentrations (1 µg/mL to 128 µg/mL). As shown in Fig. 1A, as the antibacterial protein CB6-C concentration increased, the hemolytic rate also increased slightly. At 64 µg/mL, the rate of hemolysis of sheep blood cells only reached 12.32%. These results indicated that antibacterial protein CB6-C had lower hemolytic activity. Concurrently, cytotoxicity assays also showed that RAW 264.7 cell growth was not affected by lower concentrations of antibacterial protein CB6-C (16, 32 and 64 μg/mL), and no cell death was observed at 24 h. Even though 256 μg/mL antibacterial protein CB6-C only caused 9.1% inhibition of cell growth in the RAW 264.7 cells (Fig. 1B), but the lower concentrations of antibacterial protein CB6-C did not show growth inhibition in RAW 264.7 cells. These results indicated that the proper concentration of antibacterial protein CB6-C was safe for animal use.

### Antibacterial spectrum and MIC

Table 1 show the antibacterial spectrum and MIC values of antibacterial protein CB6-C. Antibacterial protein CB6-C showed observable antimicrobial activity against gram-positive bacteria compared with gram-negative bacteria. Among these, the best antibacterial activity was shown against MRSA and *Staphylococcus*, and the lowest MIC value was 16 μg/mL. For antibacterial spectrum of the gram-negative bacteria, only the MIC of antibacterial protein CB6-C for *Escherichia coli* K88, *Klebsiella pneumoniae* CMCC(B)46,117 and *Zymomonas mobilis* ATCC29121 was 128 μg/mL, and there was no inhibitory effect against other gram-negative bacteria.

**Table 1:** Antibacterial spectrum of CB6-C

Gram reaction and strains	Source/reference		Broth medium	MIC, µg/mL
Gram-positive bacteria				
*Staphylococcus* DSO	In this study		LB	16
*Staphylococcus* N3-1	In this study		LB	16
*Staphylococcus* J101	In this study		LB	32
*Methicillin-resistant Staphylococcus aureus* (MRSA)	In this study		LB	16
*Enterococcus faecalis*	In this study		LB	> 256
*Streptococcus*	In this study		LB	32
*Bacillus cereus*	ATCC11778		LB	> 256
*Staphylococcus aureus*	ATCC 25923		LB	8
*Bacillus subtilis*	ATCC6633		LB	> 256
Gram-negative bacteria				
*Acinetobacter baumannii*	In this study		LB	> 256
*Shigella castellani*	In this study		LB	> 256
*Pseudomonas aeruginosa*	In this study		LB	> 256
*Salmonella* H9812	In this study		LB	> 256
*Escherichia coli* K88	In this study		LB	128
*Klebsiella Pneumoniae*	In this study		LB	> 256
*Klebsiella Pneumoniae*	CMCC(B)46117		LB	128
*Zymomonas mobilis*	ATCC29121		LB	128
*Escherichia coli*	ATCC 25922		LB	> 256

*ATCC* American Type Culture Collection, *CMCC(B)* China Center for Medical Culture Collections

### Time-kill kinetics

**Fig. 2:**
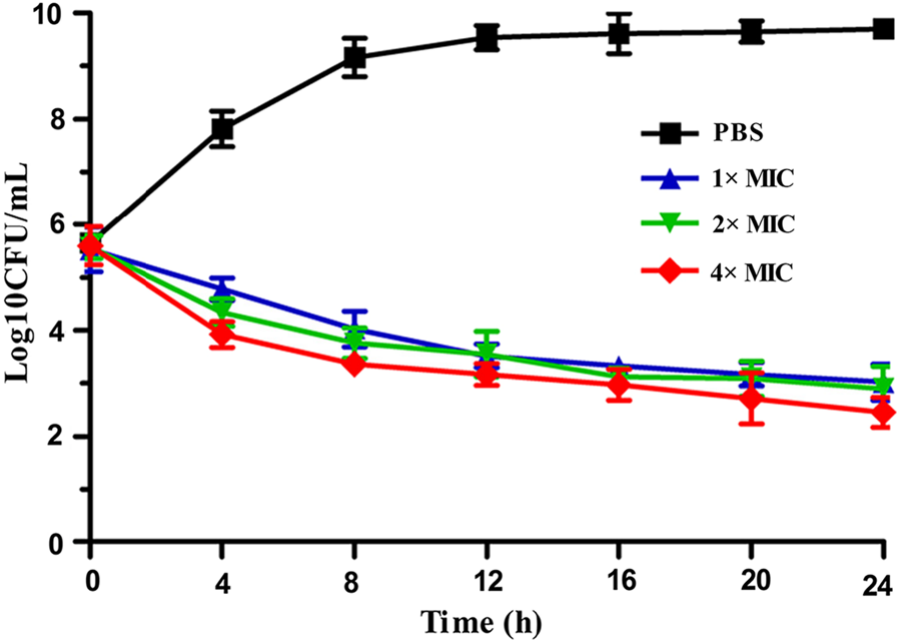
Time-kill kinetics of CB6-C

As shown in Fig. 2, the ability of different concentrations (1 × MIC, 2 × MIC and 4 × MIC) of antibacterial protein CB6-C to prevent or kill MRSA within 24 h was determined. Based on the colony counts, compared with the PBS group, the antibacterial protein CB6-C treated groups had a remarkably decreased number of bacteria at 1 × MIC. Of note, at 4 × MIC, the number of bacteria was reduced by five orders of magnitude in 24 h. These results indicated that the antibacterial protein CB6-C could inhibit bacterial growth for a long time but not thoroughly kill the bacteria.

### Stability assay

The results of the effects of temperature, pH, enzymes and organic reagents on antibacterial activity in Table 2 showed that antibacterial protein CB6-C had an optimal antibacterial activity at 40 °C to 50 °C. However, as the temperature increased, the antibacterial activity gradually decreased, and only a residual 10.1% antibacterial activity was observed at 121 °C. Different pH treatment results showed that antibacterial protein CB6-C had an optimal antibacterial activity at pH 6.0–7.0; but at low pH (2.0–5.0) the activity was slightly reduced (88–96% activity retained) while at a higher pH (10.0–12) the activity loss was the highest. Moreover, antibacterial protein CB6-C had stable antibacterial activity in the presence of catalase but lost most of the antibacterial activity in the presence of pepsin, proteinase-K, trypsin and papain. This result excludes the effect of hydrogen peroxide on the antibacterial activity of CB6-C, and shows the CB6-C presence of hydrolysis sites for pepsin, proteinase-K, trypsin and papain. We also tested the effect of organic reagents on the antibacterial activity of antibacterial protein CB6-C, and the results showed that organic reagents did not affect the antibacterial activity of antibacterial protein CB6-C.

**Table 2:** Stability of CB6-Csn after treating with thermal stability, enzymes, pH and organic reagent

Factors	Time (min)	Residual activity (%)
Temperature		
Positive control	60	100
40 °C	60	100
50 °C	60	100
60 °C	60	83.33
70 °C	60	73.33
80 °C	60	73.33
90 °C	60	60
100 °C	60	40
121 °C	15	10.10
pH		
Positive control	30	100
2	30	92
3	30	94
4	30	94
5	30	98
6	30	100
7	30	100
8	30	80
9	30	80
10	30	68
11	30	68
12	30	52
Enzymes		
Positive control	30	100
Catalase	30	100
Pepsin	30	50
Proteinase-K	30	66.67
Trypsin	30	83.33
Papain	30	66.67
Organic reagent		
Positive control	60	100
Isopropanol	60	100
Acetone	60	100
Methanol	60	100
Tween-20	60	100
Tween-80	60	100
EDTA	60	100
Acetonitrile	60	100

### Additive effect of antibacterial protein CB6-C and conventional antibiotics

The results of the antibacterial effect of CB6-C combined with other antibiotics are shown in Table 3. The combination of antibacterial protein CB6-C and ampicillin exerted a synergistic effect against MRSA. The fractional inhibitory concentration index (FICI) value was 0.28125, while the combination of antibacterial protein CB6-C with other conventional antibiotics produced an additive effect, with FICI values of from 0.5312 to 1.

**Table 3:** The anti-MRSA effect of antibacterial protein CB6-C and conventional antibiotics

Antibacterial protein (CB6-C)	Polymyxin B	Enrofloxacin	Kanamycin	Ciprofloxacin	Ampicillin	Azithromycin	Rifampin
MIC, µg/mL	8	32	128	32	64	128	128
FICI	0.5312	0.5312	0.5312	0.5312	0.28125	0.5625	1

FICI ≤ 0.5 denotes synergy and 0.5 < FICI ≤ 1.0 denotes additive

### Effect of metal ions on antibacterial protein CB6-C activity

The effect of metal ions on the antibacterial activity of antibacterial protein CB6-C is shown in Table 4. The antibacterial activity of antibacterial protein CB6-C was enhanced by K^+^, Co^2+^ and Ni^+^ among the tested metal ions, the antibacterial activity increased about 2–fourfold compared with that of the control group. However, Mg^2+^ and Mn^2+^ slightly inhibited the antibacterial activity of antibacterial protein CB6-C, while inhibition by Ca^2+^, Ba^2+^, Fe^3+^ and Co^2+^ was obvious, which showed a reduction of more than 32-fold of antibacterial activity.

**Table 4:** The anti-MRSA effect of antibacterial protein CB6-C and metal ions

Antibacterial protein	K +	Ca2 +	Ba2 +	Mg2 +	Fe3 +	Co2 +	Ni +	Mn2 +	Cu2 +
CB6-Csn	8	256	256	32	256	4	8	64	256

### ROS assay

**Fig. 3:**
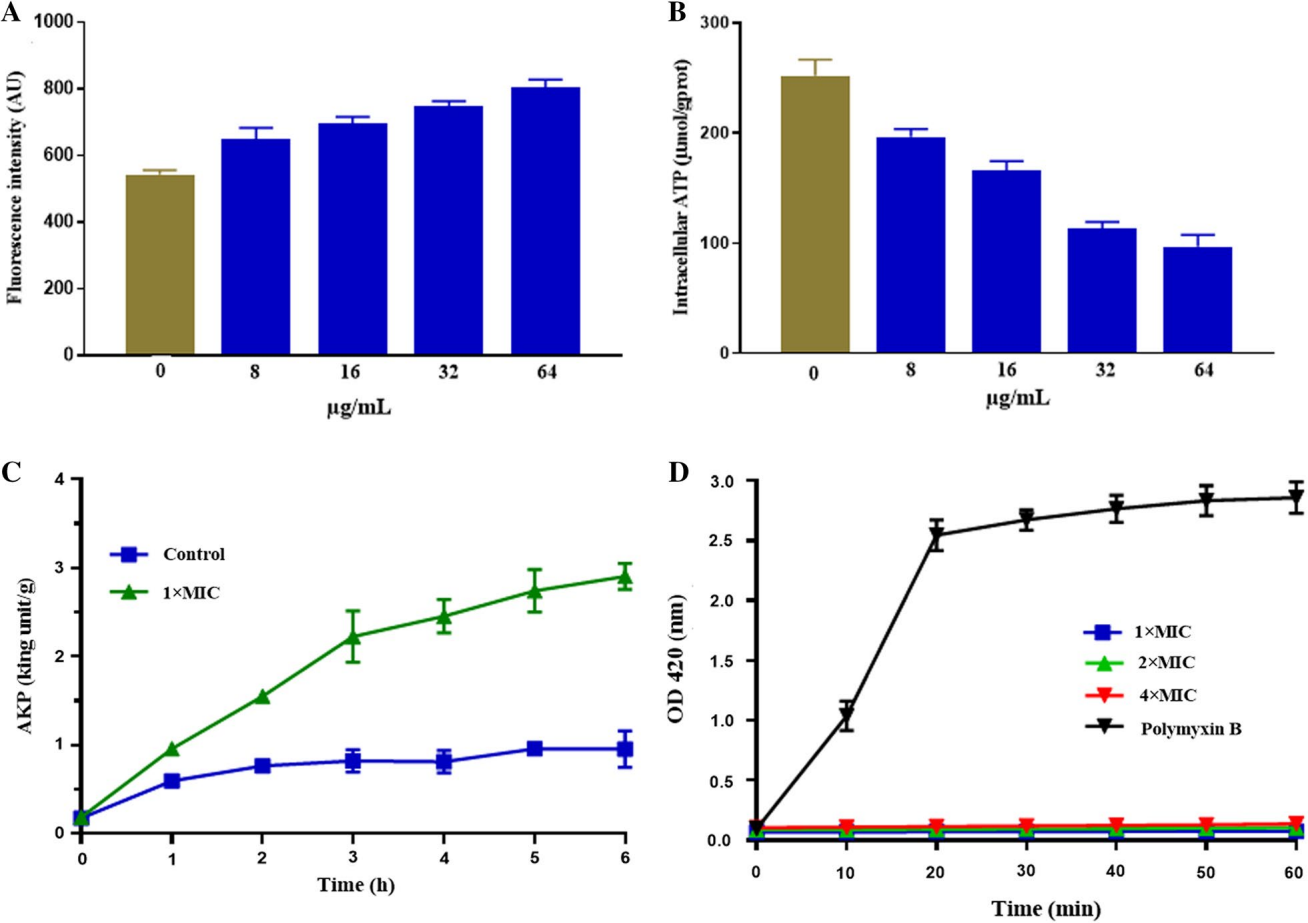
**A** Total ROS accumulation in MRSA treated with CB6-C. **B** ATP release in MRSA treated with CB6-C. **C** AKP release in MRSA treated with CB6-C. **D**. Membrane permeability of CB6-C at different concentrations

The ROS assay results are shown in Fig. 3A. At 1/2 × MIC, an antibacterial protein CB6-C obviously increased the accumulation of ROS relative to that of the untreated group and led to the accumulation of ROS in a dose-dependent manner.

### ATP and AKP release assays

Bacterial movement requires ATP to provide energy. Here, we assessed the changes in ATP after MRSA was treated with different concentrations of antibacterial protein CB6-C (8 µg/mL to 64 µg/mL). As shown in Fig. 3B, an intracellular ATP decrease in MRSA was caused by 8 µg/mL of antibacterial protein CB6-C, and as the antibacterial protein CB6-C concentration increased, the amount of intracellular ATP by MRSA largely decreased. The results showed that the higher concentration of CB6-C was more likely to cause decrease of intracellular ATP in bacteria. Additionally, AKP is an important enzyme located between the cell wall and cell membrane. When the cell wall is damaged, AKP will leak out. Therefore, AKP is commonly used as an important indicator for assessing cell wall integrity. As shown in Fig. 3C, after treatment with 1 × MIC of antibacterial protein CB6-C, the release of AKP was slightly higher than that of the control group at 1 h. With the extension of time, the release of AKP gradually increased, increased to 2.7 King units/g at 6 h.

### Membrane permeability assay

ONPG is a colorless substrate for the β-galactosidase reaction. When the cell membrane is destroyed, ONPG crosses the cell membrane and is degraded by β-galactosidase into galactose and yellow o-nitrophenol in the cell membrane. A microplate reader was used to determine the sample absorbance by the OD at 420 nm to evaluate the effect of CB6-C on the permeability of the bacterial cell membrane. The results are shown in Fig. 3D. With prolonged action time, the membrane permeability of polymyxin B increased significantly in the positive control group. However, the membrane permeability in the presence of different concentrations of CB6-C (1 × MIC, 2 × MIC and 4 × MIC) did not change. These results indicated that CB6-C had no obvious destroy effect on the cell membrane.

### Protoplasts preparation and MIC assay

As presented in Additional file 2: Fig S3, the results showed that the treated cells were all stained red, indicating that the cell wall of MRSA was completely detached, and the protoplasts of MRSA had successfully been prepared. In addition, the MIC test results showed that antibacterial protein CB6-C had no effect on the protoplasts of MRSA, which indicated that antibacterial protein mainly inhibits MRSA growth by destroying the cell wall and had no effect on the MRSA cell membrane.

### Morphological and intracellular changes in MRSA cells after treatment with antibacterial protein CB6-C

**Fig. 4:**
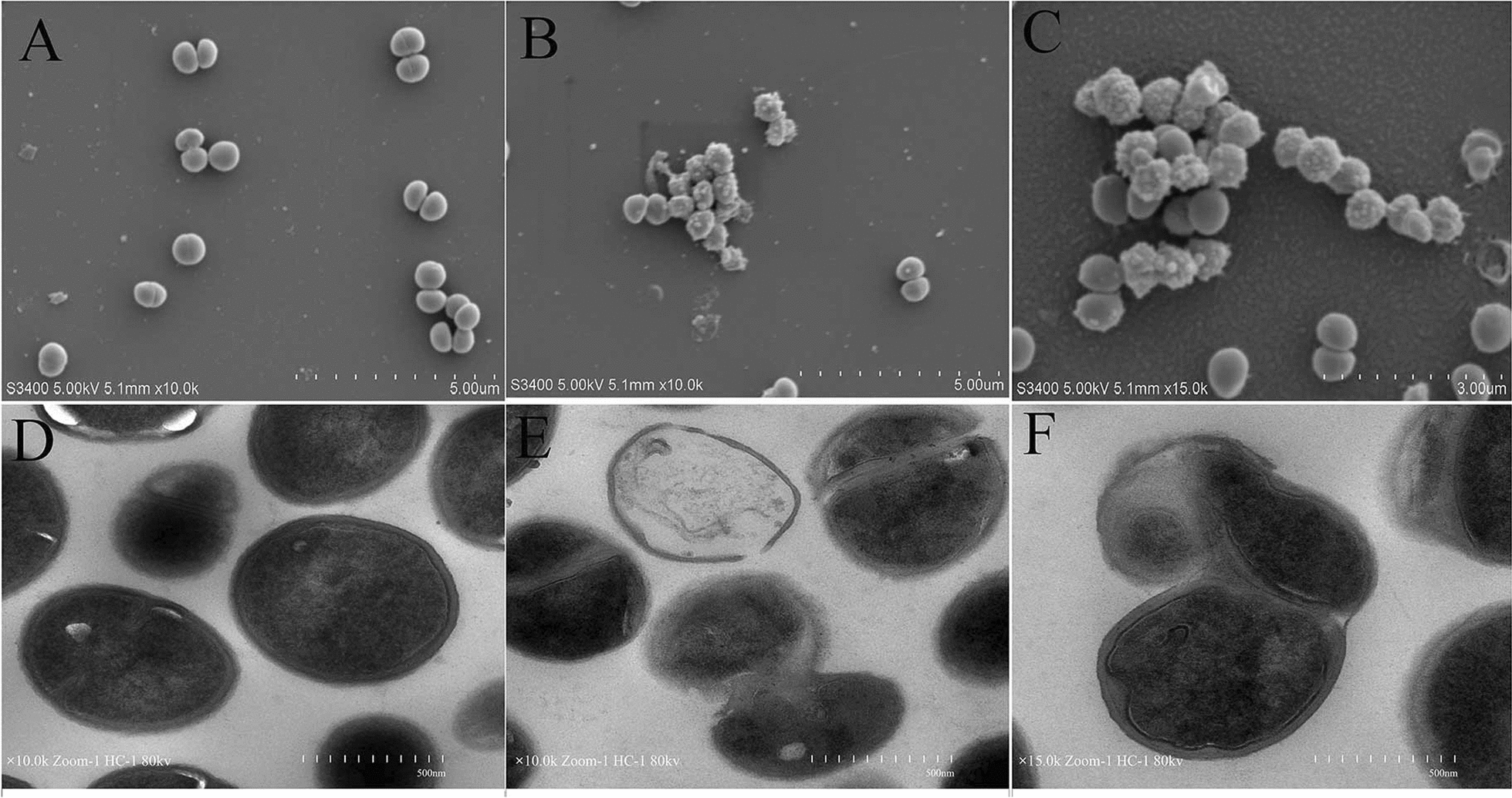
**A** Scanning electron micrographs of MRSA treated with CB6-C: **a** control; **b**, **c** CB6-C -treated. **B** Transmission electron microscopy of MRSA treated with CB6-C: **d** control; **e**, **f** CB6-C -treated

To obtain more evidence that antibacterial protein CB6-C affects MRSA, we observed bacterial morphological changes using SEM (Fig. 4A–C). MRSA cells treated with 1 × MIC of antibacterial protein CB6-C for 3 h showed many obvious vesicles on the membrane surface, and some cells were disrupted (Fig. 4B, C). In contrast, the control group exhibited intact cell structures, and the cell membrane surfaces were smooth and had plump spheres (Fig. 4A). Furthermore, intracellular changes in MRSA cells treated with antibacterial protein CB6-C were clearly observed by TEM. The TEM observation results indicated that untreated cells showed intact cell walls and cell membranes (Fig. 4D). However, after antibacterial protein CB6-C treatment of MRSA cells for 3 h, the cell wall had clearly formed pores, cytoplasm components had leaked out, and some of the cell walls had dissolved compared with that of the cells in the control group (Fig. 4E, F). Additionally, leaked cytoplasmic contents were observed around the cell. These phenomena clearly show that CB6-C inhibits the growth of MRSA by acting on the bacterial cell wall.

### Competitive inhibition assay

The effect of CB6-C on the cell wall composition of *S. aureus* was detected, and it can be inferred that the target of CB6-C was the cell wall of MRSA. As shown in Table 5, the addition of *Staphylococcal* Protein A did not affect the antibacterial activity of CB6-C against MRSA. However, after adding peptidoglycan and membrane teichoic acid, the MIC of CB6-C for MRSA increased from 16 μg/mL to 32 μg/mL and 128 μg/mL, respectively. This result indicated that the main target of CB6-C on MRSA was lipoteichoic acid on the bacterial cell wall.

**Table 5:** Effects of additional peptidoglycan, Staphylococal Protein A, and Lipoteichoic acid from on the anti-MRSA activity of CB6-C

Antimicrobial proteins (µg) Mass (µg)	MIC (μg/mL)			
	MHB	+ peptidoglycan 10 µg	+ membrane teichoic acid 10 µg	+ Staphylococal Protein A 10 µg
CB6-C	16	32	128	16

### Antibacterial protein CB6-C exhibits therapeutic efficacy in mouse models

**Fig. 5:**
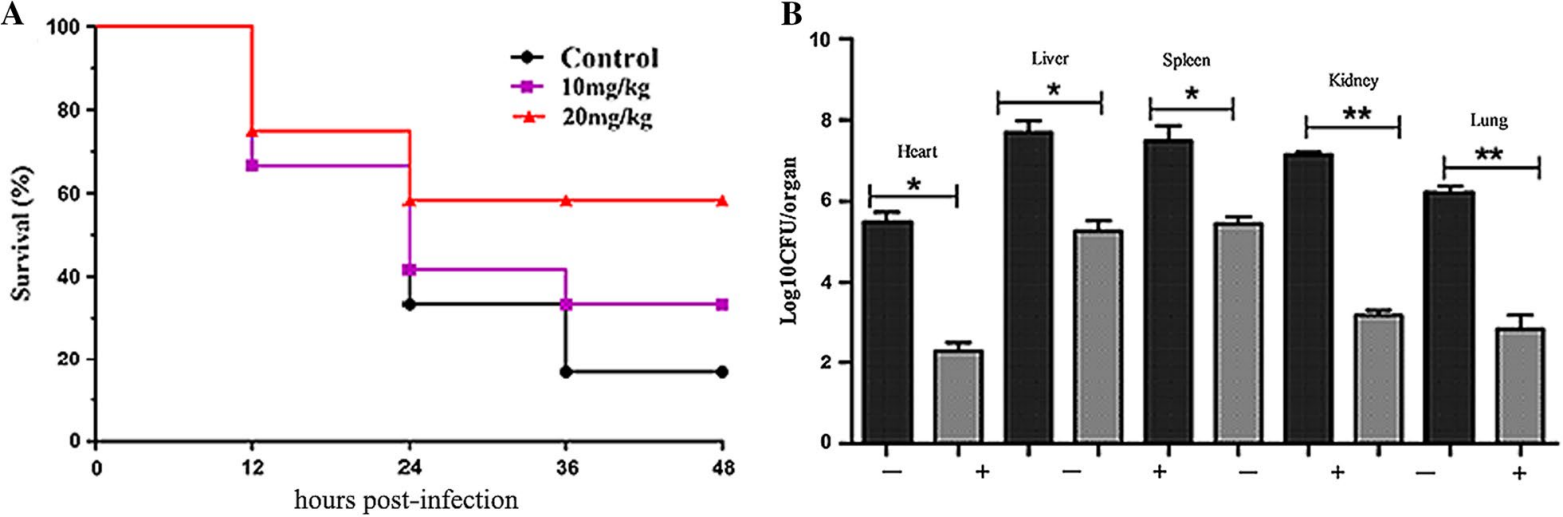
CB6-C was efficient in preventing infections. **A** Survival rates of the mice treated with CB6-C infected by MRSA (n = 10 per group). **B** Effect of CB6-C on bacterial survival in organs of mice. "−" represents the infection group, " + " represents CB6-C treatment group. Data are represented as mean ± SD. *p < 0.05, **p < 0.01, determined by non-parametric one-way ANOVA

**Fig. 6:**
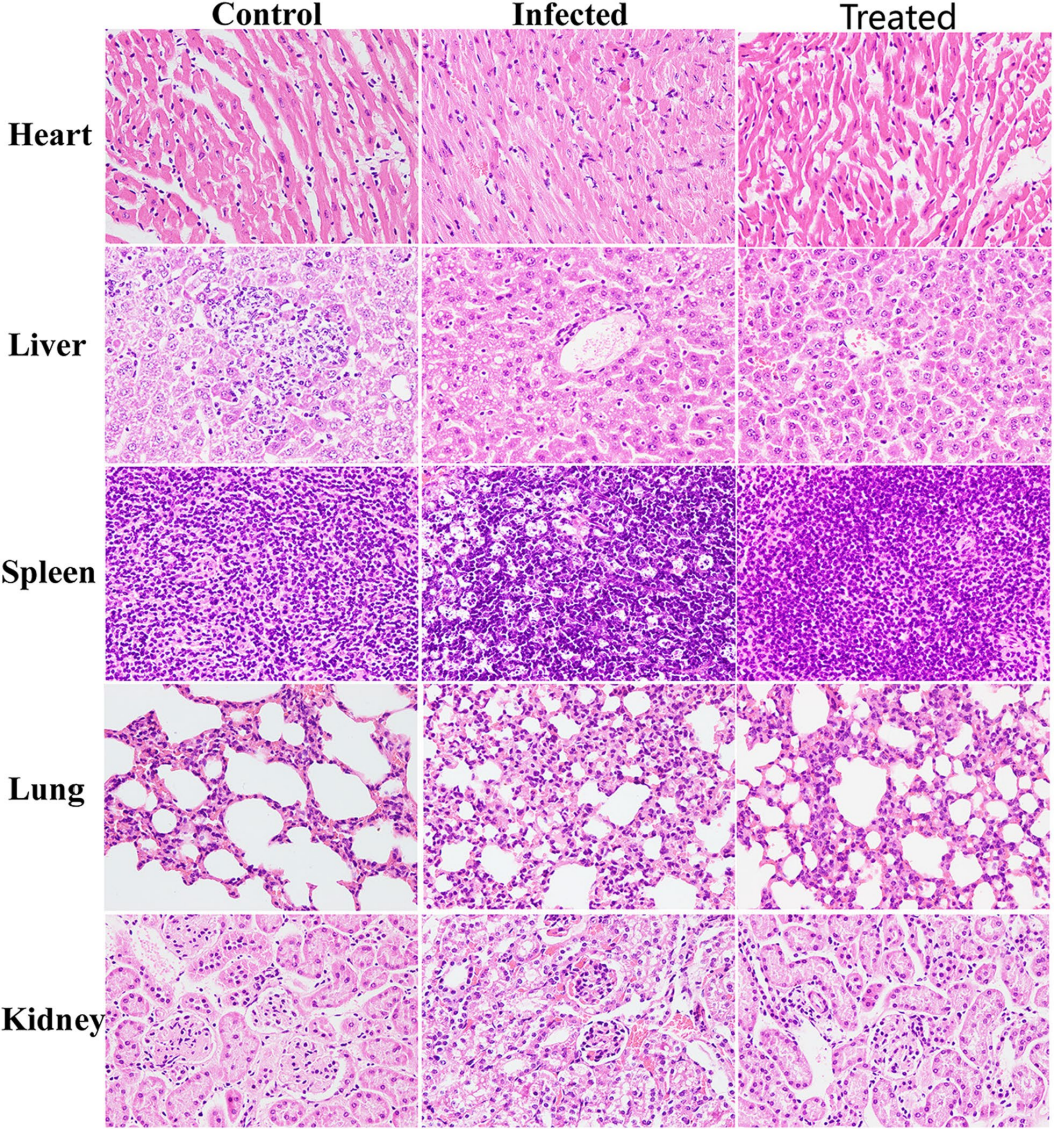
Histologic analysis of the tissues in mice using hematoxylin–eosin staining (×400)

The survival rates of mice treated with antibacterial protein CB6-C for 48 h are shown in Fig. 5A. After treatment with antibacterial protein CB6-C, the survival rates of the experimental groups were significantly increased. Among these, the survival rate of the 20 mg kg ^−1^ antibacterial protein CB6-C treated group was the highest and increased by 43% compared with that of the control group. Additionally, after 48 h of treatment in the mice, the infection group of mice had higher levels of bacteria in the spleen, liver, and kidneys and lower levels of bacteria in the heart and lungs. After CB6-C treatment, the liver and spleen bacterial contents were slightly reduced, and the kidneys, heart and lungs bacterial contents were obviously reduced (Fig. 5B). Furthermore, the HE staining results of mouse organs are shown in Fig. 6. After MRSA infection, the mouse organs were observed and extensive hemorrhage, punctate necrosis, cell hypertrophy, granular chromatin, and other pathological changes were found. After CB6-C treatment, the pathological changes of these organs had been relieved. These results show that CB6-C has a treatment effect on the damage of organs in mice.

## Discussion

Antibiotic resistance is an increasing problem globally and has become one of the crises affecting human safety. Among these resistant bacteria, MRSA can cause a variety of bacterial infections in humans and animals and lead to a series of foodborne poisoning events [20, 21]. Therefore, a safe and efficient treatment for MRSA infection is crucial to ensure human food safety and public health. In our study, we identified a new antibacterial protein CB6-C with an effective antibacterial activity against MRSA, this protein showed 88.5% identity with chitosanase (Csn) in *Bacillus subtilis* 168 (accession no. WP_044801783.1). Earlier research has shown that most of the chitosanase (Csn) proteins act on the beta-1,4-glycosidic linkage between D-glucosamine (GlcN) and N-acetyl D-glucosamine (GlcNAc) in the cell wall of fungi and have a good inhibitory effect on fungi [22]. However, there is no report on inhibiting the growth of bacteria. Therefore, this study used antibacterial protein CB6-C as a research object and analyzed the antibacterial mechanism of antibacterial protein CB6-C through a series of antibacterial tests.

To our knowledge, the hemolytic activity and cytotoxicity of antibacterial proteins are important indicators for evaluating their clinical applications [23]. Therefore, the cytotoxicity and hemolytic activity of the antibacterial protein CB6-C were tested. Among all examined concentrations, the antibacterial protein CB6-C demonstrated low levels of hemolytic activity and cytotoxicity (Fig. 1), which shows that a certain dose of antibacterial protein CB6-C was safe and nontoxic to animals. This result was consistent with a report by Muslim et al. [24] that chitosanase was safe for animals. The antibacterial spectrum results showed that antibacterial protein CB6-C had inhibitory activities against both gram-negative and gram-positive bacteria. Uniquely, an antibacterial protein CB6-C showed better antibacterial activity against gram-positive bacteria than gram-negative bacteria. Among them, the MIC of CB6-C for MRSA was 16 μg/mL. To evaluate the mode of action of antibacterial protein CB6-C on MRSA, we used a time-kill kinetics assay to reveal the relationship of antibacterial protein CB6-C concentration and time with antibacterial activity. As observed in Fig. 2, at a low concentration of antibacterial protein CB6-C, the number of bacteria remarkably decreased, and MRSA growth was restrained. Notably, at a higher concentration of antibacterial protein CB6-C, the number of bacteria was reduced by five orders of magnitude at 24 h. This indicated that exposure time and concentration can increase the antibacterial activity of antibacterial protein CB6-C but not lead to completely killing of the MRSA. Additionally, seven antibiotics from different classes were tested in combination with antibacterial protein CB6-C. We found that antibacterial protein CB6-C showed synergy with ampicillin, and the combination with other antibiotics produced an additive effect. Some studies reported that the antibacterial effect of ciprofloxacin act on DNA gyrase and polymyxin B act on the cell outer membrane, enrofloxacin inhibit bacterial DNA replication, kanamycin inhibit protein synthesis, ampicillin inhibit cell wall synthesis, azithromycin inhibit bacterial transpeptide and rifampicin inhibits RNA synthesis [25–28]. Based on these antibiotic action mechanisms, the combination of antibacterial protein CB6-C and other antibiotics improved the binding efficiency and ultimately enhanced the antibacterial activity. Therefore, an antibacterial protein CB6-C can serve as an additive to decrease the dose of antibiotics.

The stability of an antibacterial protein is the main reason affecting its clinical application [29]. To evaluate the stability of antibacterial protein CB6-C, the influence of temperature, pH, enzymes and organic reagents on antibacterial protein CB6-C was tested, and the optimal reaction temperature of antibacterial protein CB6-C was found to be 40 °C and 50 °C. These findings are in agreement with a previous study, which found that the optimal reaction temperature for chitosanase (Csn) was 40 °C and for DAU101 was 50 °C [30]. In addition, antibacterial protein CB6-C retained 60% antibacterial activity after 60 min of incubation at 90 °C. These findings showed that antibacterial protein CB6-C had high thermotolerance, and this feature was beneficial for the storage and transportation of antibacterial protein CB6-C and expanded its application prospects in other areas [31]. Furthermore, an antibacterial protein CB6-C was found to be quite pH-tolerant, the optimal pH was 6 and 7, while antibacterial protein CB6-C could maintain 90% antibacterial activity even in a strong acidic environment (pH 2.0) and 50% antibacterial activity in a strong alkaline environment. Therefore, a wide range of pH stability and high antibacterial activity under acidic conditions make antibacterial protein CB6-C a promising candidate for antibacterial drugs. Furthermore, the effects of metal ions and organic reagents on the activity of antibacterial protein CB6-C are shown in Tables 2 and 4. The antibacterial activity of antibacterial protein CB6-C was enhanced by K^+^, Co2^+^ and Ni^+^ among the metal ions. The Mg^2+^ and Mn^2+^ slightly inhibited the antibacterial activity of antibacterial protein CB6-C, while Ca^2+^, Ba^2+^, Fe^3+^ and Co^2+^ inhibition was obvious. Additionally, organic reagents such as methanol, isopropanol, and Tween 20 had no effect on the antibacterial activity of antibacterial protein CB6-C. This was consistent with the results of Zhou et al. [31], they found that many organic reagents had no effect on the activity of chitosanase (Csnm). Taken together, an antibacterial protein CB6-C had the stability characteristics of high temperature resistance, acid and alkali resistance, pH resistance and resistance to organic reagents. We believe that these characteristics make antibacterial protein CB6-C more likely to become a potential candidate for antibacterial agents.

To assess the mechanism of antibacterial protein CB6-C on MRSA, firstly we observed that antibacterial protein CB6-C reduced the intracellular ATP content in MRSA in a dose-dependent manner. In addition, antibacterial protein CB6-C triggered the accumulation of ROS in MRSA, which correspondingly aggravated bacterial damage. These findings were in agreement with a previous study, which found that endogenous ROS are crucial to kill bacteria [32]. Moreover, AKP is an important enzyme for detecting cell wall integrity. In this study, after the treatment with 1 × MIC of antibacterial protein CB6-C, the release of AKP gradually increased with time. However, membrane permeability test results showed that antibacterial protein CB6-C had no obvious effect on the bacterial cell membrane. Therefore, we hypothesized that antibacterial protein CB6-C destroyed the cell wall of bacteria to inhibit bacterial growth. For further validation of this point, we separated the protoplasts from MRSA cells and carried out MIC detection on the treated protoplasts and untreated MRSA strains. The results showed that antibacterial protein CB6-C had no inhibitory effect on the protoplasts of MRSA. We hypothesized that antibacterial protein CB6-C had no effect on the integrity of the MRSA cell membrane. In addition, the SEM results more intuitively showed that many vesicles appeared in the bacterial cell wall of the treated group. We speculated that this may be a phenomenon in which the protoplasts leaked out after the cell wall was broken. Concurrently, the TEM results clearly showed that the bacterial cell wall was obviously damaged and that dissolution occurred in a large area, while the bacteria in the control group were full and intact. It is well-documented that the biggest difference between the cell walls of Gram-positive bacteria and Gram-negative bacteria is that the Gram-positive bacteria cell walls contain a large amount of teichoic acid (Lipoteichoic acid and wall teichoic acid). Among them, one end of lipoteichoic acid is connected to the cell membrane, and the other end is free from the cell wall. Therefore, lipoteichoic acid plays an important role in resisting bacterial invasion. In addition, the cell wall of *S. aureus* also contains about 6.7% of Staphylococcus protein A. Relevant studies have shown that peptidoglycan and lipoteichoic acid on the cell wall of gram-positive bacteria are the main attack targets of some antibacterial proteins [33–36]. Therefore, peptidoglycan, lipoteichoic acid, and staphylococcus protein A are the main components on the cell wall of *S. aureus* as competitive inhibitors of CB6-C targets, which analyzed the potential active site of antibacterial protein CB6-C on the cell wall of MRSA. The results show that the addition of lipoteichoic acid can effectively reduce the antibacterial activity of CB6-C against MRSA. Therefore, we speculated that the target of antibacterial protein CB6-C may be on the lipoteichoic acid of the cell wall in gram-positive bacteria. In this context, antibacterial protein CB6-C had a good inhibitory effect on a variety of gram-positive bacteria, providing a reasonable explanation. Taken together, these results suggest that antibacterial protein CB6-C can kill bacteria by destroying the lipoteichoic acid of the bacterial cell walls.

Given the effective antibacterial activity of antibacterial protein CB6-C against MRSA in vitro, we further investigated its potential as a treatment agent in in vivo animal models. In the treatment test after mice were infected with MRSA, the mice treated with 20 mg kg^−1^ antibacterial protein CB6-C showed strikingly increased survival rates (43%) in the MRSA infection model, superior to the group treated with 10 mg kg^−1^ antibacterial protein CB6-C (Fig. 5). Consistently, the bacterial load in the different organs of the mice was significantly reduced in the antibacterial protein CB6-C treated group compared with the infection group, and after CB6-C treatment, the pathological changes of various organs were obviously restored to normal levels and had a therapeutic effect (Fig. 6). These findings together demonstrated the potential of antibacterial protein CB6-C as an alternative to antibiotics and its potential for usage against MRSA in vivo.

## Conclusions

In summary, we used a series of protein purification methods to identify the antimicrobial protein obtained from *Bacillus velezensis* CB6 as antibacterial protein CB6-C, which was shown to have the MIC of 16 μg/mL for MRSA. Analysis of the biological characteristics showed that antibacterial protein CB6-C had good stability and safety and demonstrated an additive effect with conventional antibiotics and metal ions such as Co^2+^, Ni^+^ and K^+^. The antibacterial mechanism study demonstrated that antibacterial protein CB6-C primarily destroyed the integrity of lipoteichoic acid on the bacterial cell wall, ultimately led to bacterial death. In addition, an antibacterial protein CB6-C was efficient in decreasing MRSA infections in in vivo models. These results indicated that antibacterial protein CB6-C has the potential to become an antibacterial agent.

## Supplementary Information

**Additional file 1: Table S1**. Bacteria strains used in this study

**Additional file 2: Fig S1.** (A) Sephadex G-75 elution profile of antibacterial protein CB6-C. (B) QAE-Sephadex A25 elution profile of antibacterial protein CB6-C. **Fig S2.** (A) HPLC elution profile of antibacterial protein CB6-C; (B) SDS-PAGE of antibacterial protein CB6-C. Lane M: high molecular marker, lane 1–3: Stained of the gel showing purified antibacterial protein CB6-C. **Fig. S3.** (A) Gram-stained photograph after MRSA has removed the cell wall. (B). Gram-stained photograph of untreated cells for MRSA.

## Data Availability

The datasets used and analyzed during the current study are available from the corresponding author on reasonable request.
