# Supplementary material for: Discovery of a novel antibacterial protein CB6-C to target methicillin-resistant Staphylococcus aureus
Source: Microb Cell Fact. 2022 Jan 4;21:4. doi: 10.1186/s12934-021-01726-9 (PMC8725309; doi:10.1186/s12934-021-01726-9)
Supplement: Supplementary file 1 — Additional file 1: Table S1. Bacteria strains used in this study [file 12934_2021_1726_MOESM1_ESM.docx]

| Gram reaction and strains | Source/Reference | Broth medium |
| --- | --- | --- |
| **Gram-positive bacteria** | |  |
| *Staphylococcus* DSO | In this study | LB |
| *Staphylococcus* N3-1 | In this study | LB |
| *Staphylococcus* J101 | In this study | LB |
| *Methicillin-resistant Staphylococcus aureus* (MRSA) | In this study | LB |
| *Enterococcus faecalis* | In this study | LB |
| *Streptococcus* | In this study | LB |
| *Salmonella* H9812 | In this study | LB |
| *Bacillus cereus* | ATCC11778 | LB |
| *Staphylococcus aureus* | ATCC 25923 | LB |
| *Bacillus subtilis* | ATCC*6633* | LB |
| **Gram-negative bacteria** | |  |
| *Acinetobacter baumannii* | In this study | LB |
| *Shigella castellani* | In this study | LB |
| *Pseudomonas aeruginosa* | In this study | LB |
| *Escherichia coli* K88 | In this study | LB |
| *Klebsiella Pneumoniae* | In this study | LB |
| *Klebsiella Pneumoniae* | CMCC(B)46117 | LB |
| *Zymomonas mobilis* | ATCC29121 | LB |
| *Escherichia coli* | ATCC 25922 | LB |

**Supplementary Tables**

**Supplementary Table 1**. Bacteria strains used in this study

ATCC, American Type Culture Collection; CMCC(B), China Center for Medical Culture Collections;
