# Supplementary material for: Discovery of a novel antibacterial protein CB6-C to target methicillin-resistant Staphylococcus aureus
Source: Microb Cell Fact. 2022 Jan 4;21:4. doi: 10.1186/s12934-021-01726-9 (PMC8725309; doi:10.1186/s12934-021-01726-9)
Supplement: Supplementary file 2 — Additional file 2: Fig S1. (A) Sephadex G-75 elution profile of antibacterial protein CB6-C. (B) QAE-Sephadex A25 elution profile of antibacterial protein CB6-C. Fig S2. (A) HPLC elution profile of antibacterial protein CB6-C; (B) SDS-PAGE of antibacterial protein CB6-C. Lane M: high molecular marker, lane 1–3: Stained of the gel showing purified antibacterial protein CB6-C. Fig. S3. (A) Gram-stained photograph after MRSA has removed the cell wall. (B). Gram-stained photograph of untreated cells for MRSA. [file 12934_2021_1726_MOESM2_ESM.docx]

**
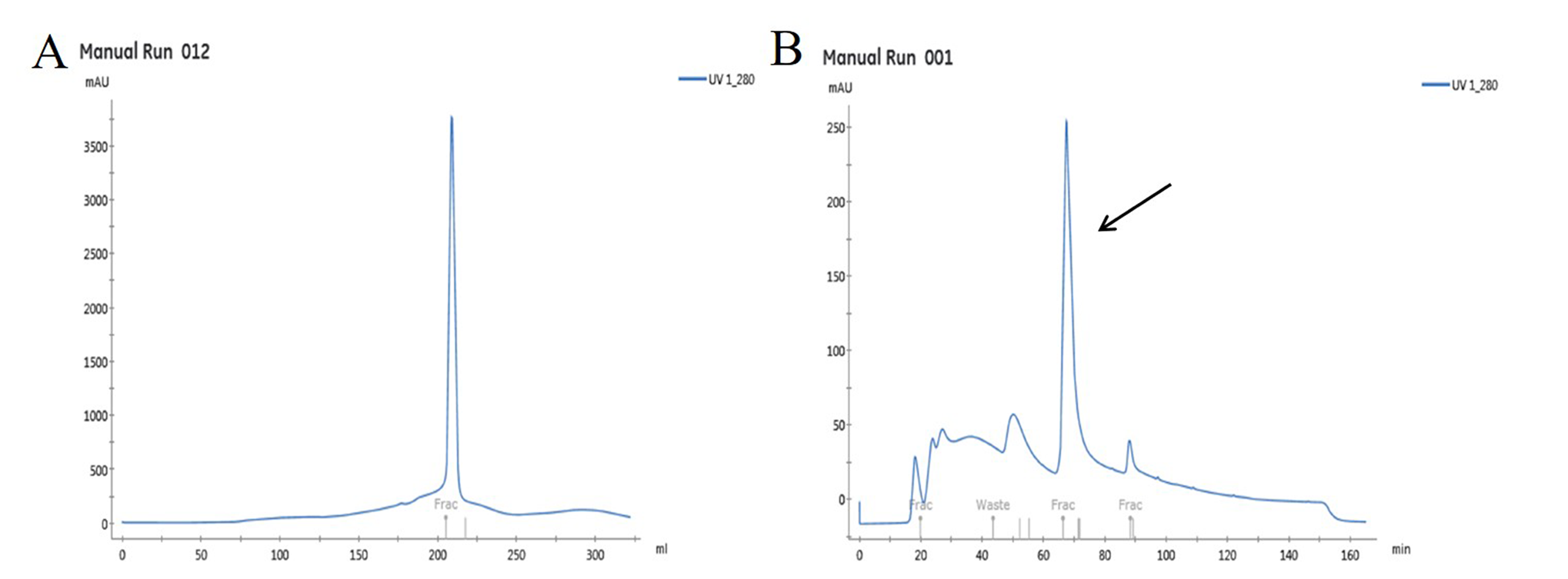
Supplementary Fig captions**

**Supplementary Fig 1.** (A) Sephadex G-75 elution profile of antibacterial protein CB6-C. (B) QAE-Sephadex A25 elution profile of antibacterial protein CB6-C.

**
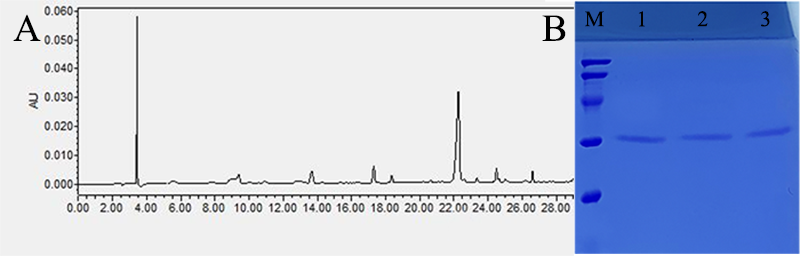
**

**Supplementary Fig 2.** (A) HPLC elution profile of antibacterial protein CB6-C; (B) SDS-PAGE of antibacterial protein CB6-C. Lane M: high molecular marker, lane 1-3: Stained of the gel showing purified antibacterial protein CB6-C.


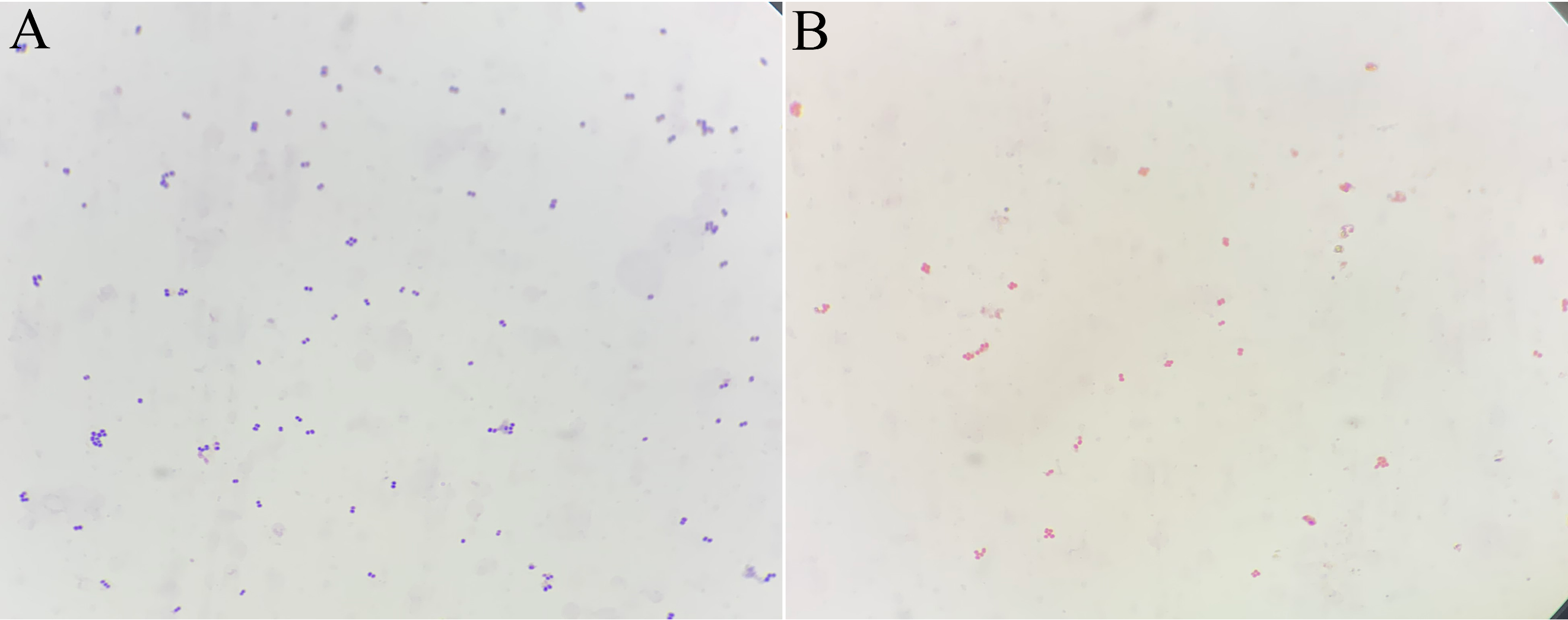


**Supplementary Fig 3.** (A) Gram-stained photograph after MRSA has removed the cell wall. (B). Gram-stained photograph of untreated cells for MRSA.
